# Effect of arginine on proliferation of porcine ovarian granulosa cells and steroid hormone synthesis

**DOI:** 10.3389/fvets.2026.1766335

**Published:** 2026-02-04

**Authors:** Tianrui Zhang, Xintan Lu, Xinyi Wang, Zebei Liu, Xuwen Shao, Rui Han, Dongsheng Che

**Affiliations:** Key Laboratory of Animal Production, Product Quality and Security, Ministry of Education, Jilin Provincial Key Laboratory of Animal Nutrition and Feed Science, College of Animal Science and Technology, Jilin Agricultural University, Changchun, China

**Keywords:** cell proliferation, granulosa cell, ovary, pig, steroid hormone

## Abstract

Granulosa cells of the ovary (GOC) served as a core component of the follicular microenvironment, playing a crucial role in regulating steroid hormone synthesis and maintaining pregnancy initiation during key reproductive processes. Arginine (Arg) was a key amino acid in follicular fluid that played a potential role in regulating animal reproduction. However, the mechanism by which it controlled the secretion of steroid hormones by ovarian granulosa cells during the initiation of pregnancy remained unclear. In this study, primary porcine ovarian granulosa cells were used to investigate the effects of arginine on cell proliferation, steroid hormone synthesis, and the underlying molecular mechanisms related to pregnancy maintenance. Results showed that the average arginine concentration in follicles measuring 3–8 mm in diameter was 2.7 mM. Based on these measurements, five arginine supplementation groups were established. Findings indicated that the 4.2 mM arginine group exhibited the highest granulosa cell proliferation efficiency, significantly higher than the control group (*p* < 0.01). The 4.2 mM arginine group significantly upregulated the expression of genes related to cell proliferation, cell cycle, and apoptosis. Concurrently, the 4.2 mM arginine group upregulated the expression of steroid hormone synthesis-related genes *CYP11A1* (*p* < 0.01) and *STAR* (*p* < 0.01), thereby promoting estradiol synthesis. Transcriptome sequencing revealed the molecular mechanism by which arginine promoted proliferation and hormone secretion in porcine ovarian granulosa cells. The study found that arginine significantly upregulated the expression of *STARD3* and *STARD6*, key genes in the cholesterol metabolism pathway. This study underscored the pivotal function of arginine in the regulation of reproduction, elucidating its influence on cellular cholesterol metabolism, the promotion of porcine ovarian granulosa cell proliferation, the inhibition of apoptosis, the enhancement of cellular activity, and the facilitation of steroid hormone synthesis through the mediation of *STARD3* and *STARD6*.

## 1 Introduction

In the context of large-scale pig farming operations, enhancing the reproductive performance of sows is pivotal to optimizing economic returns. Moreover, pigs are increasingly recognized as valuable large-animal models for biomedical and translational research because of their close similarity to humans in physiology, metabolism, and endocrine regulation, particularly in reproductive systems (1). Granulosa cells, a vital component of the ovary, play an indispensable role in follicular development, oocyte maturation, and steroid hormone synthesis. These cells establish regulatory networks with oocytes, thereby establishing a microenvironment and providing nutrients essential for their development. Additionally, the synthesis and secretion of steroid hormones play a pivotal role in the reproductive cycle, follicular development, and ovulation in sows (2). Consequently, ovarian granulosa cells function as the fundamental structural elements of the follicular microenvironment, thereby playing a pivotal role in the reproductive process of female mammals (3).

The synthesis of estradiol is primarily catalyzed by cytochrome P450 aromatase (encoded by the *CYP19A1* gene) in granulosa cells, which converts androgens into estradiol (4, 5). During the initial phases of follicular development, gonadotropins, including follicle-stimulating hormone (FSH), bind to receptors located on the surface of granulosa cells, thereby activating a series of signaling pathways. Consequently, this results in the upregulation of *CYP19A1* gene expression, thereby promoting the synthesis and secretion of estradiol (6). During the early stages of follicular development, gonadotropins, such as follicle-stimulating hormone (FSH), bind to receptors on the surface of granulosa cells. This activates a series of signaling pathways. This subsequently increases the expression of the CYP19A1 gene, which promotes the synthesis and secretion of estradiol (7, 8). During the early stages of follicular development, gonadotropins, such as follicle-stimulating hormone (FSH), bind to receptors on the surface of granulosa cells. This activates a series of signaling pathways. This subsequently increases the expression of the CYP19A1 gene, which promotes the synthesis and secretion of estradiol (9). The hormones secreted by the gonadotropin-releasing hormone (GnRH) in pigs are finely regulated by multiple factors. For example, an adequate supply of nutrients such as amino acids, fatty acids and vitamins provides the necessary substrates and energy for hormone synthesis, thereby promoting the secretion of estradiol (10, 11).

Arg has become a research hotspot due to its unique biological characteristics and physiological functions. Not only does it participate in protein synthesis, it also engages in various metabolic processes within the body, such as nitric oxide synthesis. It also has the potential to regulate cell growth, survival and endocrine functions (12). Arginine plays a crucial role in the development of the reproductive organs of female mammals. Research indicates that, in mouse experiments, supplementing the diet with arginine during pregnancy significantly promotes the development of the uterus and ovaries in female offspring (13). Histological examination revealed that the myometrium of the uterus thickened in the group of mice treated with arginine (14, 15), while the ovaries exhibited an increased number of mature follicles (16). Research on sheep ovarian cells has revealed that arginine promotes cell proliferation and survival, as well as enhancing cellular resistance to oxidative stress (17). Related studies on pigs have also reported that adding arginine can improve the environment for embryonic development and enhance embryo quality (18). Research indicates that arginine has a significant influence on the balance between the proliferation and apoptosis of granulosa cells in follicles. In experiments involving the *in vitro* culture of porcine follicular granulosa cells, adding an appropriate amount of arginine was found to significantly accelerate the cells’ proliferation rate (19). Further studies revealed that arginine may promote cell proliferation by activating relevant signaling pathways, such as the PI3K/Akt pathway, thereby enhancing cyclin expression and facilitating the transition from the G1 phase to the S phase (19). Conversely, in cases of arginine deficiency, there is a significant increase in the apoptosis rate of granulosa cells in follicles, as evidenced by heightened activity of apoptosis-related proteins such as Caspase-3 (20). This finding suggests that arginine plays a pivotal role in preserving granulosa cell viability and counteracting excessive apoptosis. However, there is a paucity of in-depth research on the specific effects of arginine on porcine ovarian granulosa cell activity, particularly regarding the molecular mechanisms by which arginine influences steroid hormone synthesis in porcine ovarian cells. This knowledge gap has restricted the practical implementation of nutritional regulation strategies to enhance sow reproductive performance.

The extant research suggests that arginine has the capacity to influence the supply of steroid hormone precursors by regulating key enzymes in cholesterol metabolism, such as CYP19A1 and STAR (21). STARD3 and STARD6 represent pivotal constituents of the steroid-activated regulator of lipid transport (StAR) family of lipid transporters. These enzymes are responsible for the transport of cholesterol during the synthesis of steroid hormones, facilitating the transfer of cholesterol from the outer membrane to the mitochondria. This process provides the necessary raw materials for steroid hormone synthesis and occupies a pivotal position in the steroid hormone biosynthetic pathway (22). As demonstrated in previous studies conducted on other species, changes in the expression of STARD3 and STARD6 are closely associated with the regulation of steroid hormone synthesis (23). However, in porcine ovarian cells, the molecular pathway by which arginine regulates cholesterol transport through mediating STARD3 and STARD6 expression, thereby influencing steroid hormone synthesis, remains unclear. Furthermore, although the beneficial effects of L-arginine on mammalian reproductive performance have been extensively documented (24), its underlying mechanisms remain poorly understood. This lack of clarity limits the precise application of arginine in regulating reproductive function in mammals such as pigs. While the research team’s prior studies revealed that arginine can modulate early embryonic development in pigs by regulating zygotic genome activation, cellular energy metabolism, and oxidative stress homeostasis (25). However, systematic studies on how its regulation of GOC proliferation indirectly influences the hormone synthesis microenvironment and its dose–response relationship during the initiation phase of pregnancy maintenance remain lacking. In the porcine GOC, the differential effects of Arg concentration changes on key P4 synthesis enzymes, such as 3β-Hydroxysteroid Dehydrogenase Type 1 (HSD3B1), as well as the interactions between the NO signaling pathway and hormone synthesis pathways, require in-depth analysis.

The present study focuses on the core physiological process of steroid hormone synthesis by identifying the effects of Arg on porcine GOC function. Utilizing an *in vitro* culture model combined with transcriptomic sequencing, ELISA, and RT-PCR techniques, the present study investigates Arg’s regulatory effects on cholesterol metabolic pathways, key enzyme activities, and hormone secretion within the GOC. The objective of this study is to elucidate the role of the Arg-mediated STARD3/STARD6 signaling axis in steroid hormone synthesis, thereby revealing its potential regulatory mechanism in initiating pregnancy maintenance. This research provides a novel perspective on the refinement of the theory of reproductive nutrition regulation in mammals.

## 2 Materials and methods

### 2.1 Detection of arginine content in follicular fluid

Follicular fluid was extracted and separated from follicles of varying diameters within porcine ovarian tissue samples obtained from a local slaughterhouse. Following centrifugation and filtration, the fluid was stored at −80 °C. Following pretreatment of porcine follicular fluid amino acid samples, liquid chromatography (HPLC) was employed to analyse arginine content in multiple batches of pooled follicular fluid samples (each containing >10 follicles) from pig ovaries. The samples comprised follicular fluid from follicles measuring less than 3 mm in diameter and those ranging from 3 to 8 mm in diameter, followed by data analysis.

### 2.2 Cell culture and identification of porcine ovarian granulosa

Primary porcine ovarian granulosa cells were isolated from porcine ovarian tissue samples using syringes. Following centrifugation in a Phosphate Buffered Saline (PBS) buffer (SH30256.01, HyClone, Cytiva, Logan, UT, USA), the upper layer of the sample was discarded, and the lower layer, i.e., the pellet, was collected. Subsequently, cells were transferred to complete medium based on DMEM/F12 (11,330,032, Gibco, Thermo Fisher Scientific, Waltham, MA, USA), containing 10% fetal bovine serum (C04001, Biological Industries, Kibbutz Beit-Haemek, Israel), 1% dual antibiotics (15140–122, penicillin–streptomycin, Gibco, Thermo Fisher Scientific, Waltham, MA, USA), gentamicin (G1264, Sigma-Aldrich, St. Louis, MO, USA), and amphotericin B (B540721, Shanghai Santong Biotechnology Co., Ltd.). Following the uniform dispersion of cells in the medium, the cell suspension was transferred to culture vessels and incubated statically at 37.5 °C, 5% CO₂, and saturated humidity. When cell confluence reaches 80%, fix with 4% paraformaldehyde, permeabilize with 0.2% Triton X-100 for 30 min, block with 5% BSA for 1 h, primary antibody FSHR (Follicle-Stimulating Hormone Receptor) (22665-1-AP,1:200, Proteintech Group, USA) overnight at 4 °C, fluorescent secondary antibody (SA00003-2,1:1,000, Proteintech Group, USA) for 1 h in the dark, DAPI for 5 min in the dark, and observation and identification of isolated porcine ovarian granulosa cells under a fluorescence microscope. When cell density in flasks reaches over 80%, the digest and passage of cells should be undertaken using 0.25% trypsin–EDTA (25,200,072, Gibco, Thermo Fisher Scientific, Waltham, MA, USA) in order to prepare a single-cell suspension. The reculture process was then repeated under identical conditions. During the culturing process, it is imperative to minimize movement of the flask in order to ensure the maintenance of a stable growth environment. It is imperative to undertake regular observations of cell growth status, meticulously documenting the progression of proliferation.

### 2.3 Detection of granulosa cell proliferation in pig ovaries

Isolate and culture porcine ovarian granulosa cells from porcine ovarian tissue. Primary porcine ovarian granulosa cells were cultured in complete medium, with DMEM/F1 serving as the control group. Five additional arginine-treated groups were established with concentrations of 2.1 mM, 2.7 mM, 3.2 mM, 3.7 mM, and 4.0 mM (A5006, Sigma-Aldrich, St. Louis, MO, USA). The experiment was performed with a minimum of three valid replicates. The RTCA (Real Time Cellular Analysis) instrument was utilized to ascertain the effect of Arg levels on the proliferation of primary porcine ovarian granulosa cells cultured *in vitro*. In the E-Plate 96-well plate compatible with the RTCA instrument, 100 μL of cell suspension adjusted to the appropriate density was added to each well, resulting in a cell seeding density of 5 × 10^3^ cells per well. Subsequent to the process of seeding, the 96-well plate was carefully transferred into the RTCA instrument’s detection chamber. The RTCA instrument and its associated software were activated, with detection parameters set. The detection frequency was configured to measure every 15 min for a continuous 120-h monitoring period, enabling real-time tracking of cellular proliferation dynamics. Subsequent to the 120-h detection period, the exportation of cell proliferation curve data from the RTCA software is required, inclusive of the electrical impedance values (Cell Index values) for each well at varying time points. Thereafter, the cell proliferation curve should be plotted.

### 2.4 Detection of apoptosis in porcine ovarian granulosa cells

The next step is to seed 3 × 10^6^ cells into each large culture dish. Following cell adhesion, the addition of 4.2 mM arginine (A5006, Sigma-Aldrich, St. Louis, MO, USA) is required, followed by incubation for a period of 72 h. Thereafter, the culture medium should be discarded. Subsequently, trypsin should be added to digest the cells (25,200,072, Gibco, Thermo Fisher Scientific, Waltham, MA, USA) and prepare a single-cell suspension. Centrifugation at a temperature of 4 °C and a speed of 1,000 g for a duration of 3–5 min is then required, after which the upper layer must be discarded. The cells should then be resuspended in 1 mL of pre-chilled PBS (SH30256.01, HyClone, Cytiva, Logan, UT, USA). The samples should then be subjected to a centrifugal process at a temperature of 4 °C and at a rotational speed of 1,000 r/min for a period of 3–5 min. The initial step in this procedure is the discarding of the cell suspension, with the cell pellet then being retained. The cells should then be resuspended in 200 μL of 1 × Binding Buffer, ensuring that all cells are retained. The cell suspension (comprising a minimum of 1 × 10^6^ cells) should be transferred to a 5 mL flow cytometry tube (hereafter referred to as the ‘tube’). Subsequently, 5 μL of FITC Annexin V (556,547, BD Biosciences, San Jose, CA, USA) and 5 μL of PI should be added. The cells should be gently vortexed and then incubated at room temperature (25 °C) in the dark for a period of 15 min, with the mixture being inverted and mixed at intervals of between 3 and 5 min. Subsequently, 200 μL of 1 × Binding Buffer should be added to each tube, following which the tubes should be analysed by flow cytometry.

### 2.5 Steroid hormone content

Primary porcine ovarian granulosa cells were cultivated in complete medium based on DMEM/F12: control group (0.62 mM Arg) and treatment group (4.2 mM Arg), with each experiment conducted in triplicate or more. Following a 72-h *in vitro* culture period, the cell culture medium was collected and the concentration of the steroid hormone (estradiol) was measured in the medium using an ELISA assay (ML002366,mlbio, Shanghai, China).

### 2.6 RT-qPCR

Primary porcine ovarian granulosa cells were cultured in a complete medium consisting of DMEM/F12 supplemented with either 0.62 mM or 4.2 mM arginine. The experiment was conducted in triplicate or more. After 72 h of *in vitro* culture of porcine ovarian granulosa cells, the cells were harvested and total RNA was extracted. Total RNA was extracted using TRIzol reagent (12204-01, Life Technologies, Thermo Fisher Scientific, USA) according to the manufacturer’s instructions. RNA concentration and purity were assessed using a NanoDrop spectrophotometer. The RNA was then reverse transcribed into cDNA using a reverse transcription kit (AT311, TransGen Biotech, Beijing, China). qPCR was performed using *β*-actin as an internal reference with SYBR Green qPCR master mix (AQ601, TransGen Biotech, Beijing, China) to detect cell proliferation (*PCNA, CDK1*, *CCND1*, *CCNB1*), apoptosis (*BCL2*, *BAX*, *CASP3*), steroid hormone synthesis (*CYP11A1*, *CYP19A1*, *STAR*) and cholesterol transport (*STARD3*, *STARD6*), with the sequences provided in Table 1.

**Table 1:** Primers for real-time quantitative PCR.

Gene name	GeneBank number	Primer sequence(5′-3′)
*β-actin*	XM_021086047.1	F: CATCCTGCGTCTGGACCTGG R: TAATGTCACGCACGATTTCC
*PCNA*	NM_001291925.1	F: GGCTCTATCCTGAAGAAGGTGCTG R: GACATGAGACGAGTCCATGCTCTG
*CDK1*	NM_001159304.2	F: AACCACTTTTCCACGGGGATTCAG R: GCTAGGCTTCCTGGTTTCCACTTG
*CDK4*	NM_001123097.1	F: CGGAGATTGGTGTTGGTGCCTATG R: CCATTGGGGACTCTTACGCTCTTG
*CCND1*	XM_021082686.1	F: GCTCGCCCTCCGTGTCCTAC R: TCGCAGACCTCCAGCATCCAG
*CCNB1*	NM_001170768.1	F: GCCCTCTACCCCTGCATTTTCTTC R: GCTGCGATCTGAGAAGGAGGAAAG
*BCL2*	XM_021099593.1	F: GATGCCTTTGTGGAGCTGTATGG R: CCAGGGCCAGACTGAGCAG
*BAX*	XM_003127290.5	F: ATCGGCTGCTGGGCTGGATC R: ATGGTGAGCGAGGCGGTGAG
*CASP3*	NM_214131.1	F: GCTGTAGAACTCTAACTGGCAAACC R: CCCACTGTCCGTCTCAATCCC
*CYP11A1*	NM_214427.1	F: CCTGGTGACAATGGCTGGATTAAC R: GGAAGTTCTGGACATGGTGATAGTG
*CYP19A1*	NM_214429.1	F: TCATAAGAGTCTGGATAGGTGGAGAAG R: GCTGCCAAATCGGGATGTGTAG
*STAR*	NM_213755.3	F: GCTGGAGGTGGTGGTGGAC R: TGCAGGATCTTGATCTTCTTGACAC
*STARD3*	NM_001143725.1	F: GAAGAACTTGGAGCAGGAGATCATC R: GCCGCAGCACAGCATAGC
*STARD6*	XM_013992874.2	F: CTTCAAGTTACATCCGTGGTTACAAC R: CTCATTTCTGTCTGGACAAACATCAC

### 2.7 Transcriptome sequencing

The present study employed *in vitro* cultured porcine ovarian granulosa cells to construct six RNA-seq libraries, following the Illumina standard workflow. Total RNA was extracted from porcine ovarian granulosa cells using an RNA extraction kit (12204-01, Life Technologies, Thermo Fisher Scientific, USA), according to the manufacturer’s instructions. Briefly, cells were collected after treatment and lysed using the lysis buffer provided in the kit to release total RNA. The lysates were processed following the standard protocol supplied by the manufacturer to remove proteins and other contaminants. Total RNA was finally eluted in RNase-free water. The concentration and purity of RNA were determined by measuring absorbance at 260 and 280 nm, and the extracted RNA was stored at −80 °C until further use for reverse transcription and RT-qPCR analysis. First, mRNA was enriched using Oligo(dT) magnetic beads and fragmented into short pieces in fragmentation buffer. Subsequently, random primers were employed to synthesize first- and second-strand cDNA templates. Following magnetic purification, end repair, A-tailing, and adapter ligation were performed. Fragments were screened again, and PCR amplification yielded complete libraries. Sequencing was completed on the Illumina platform by Wuhan Maiwei Metabolic Biotechnology Co., Ltd. Differentially expressed genes were identified by employing the DESeq approach (version 1.38.3) with thresholds set at |log₂FoldChange| > 1 and *p* < 0.05. Subsequent pathway enrichment analysis was conducted against the KEGG database, with significant pathways determined through hypergeometric testing (*p* < 0.05).

### 2.8 Statistical analysis

One-way analysis of variance (ANOVA) was performed using SPSS 27.0 software to assess differences between groups. Graphs were generated using GraphPad Prism 9.5 software. Results are expressed as mean ± standard error. *p* < 0.05 indicates significant differences. *p* < 0.01 indicates extremely significant differences.

## 3 Results

### 3.1 Detection of arginine content in porcine follicular fluid and identification of porcine ovarian granulosa cells

**Figure 1:**
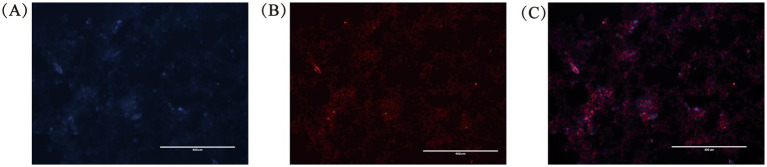
FSHR immunofluorescence staining of granular cells isolated and cultured in vitro. **(A)** Nuclei stained with propidium iodide (PI), showing nuclear distribution of granulosa cells. **(B)** Immunofluorescence staining of follicle-stimulating hormone receptor (FSHR) in granulosa cells. **(C)** Merged image of PI and FSHR staining, showing co-localization of nuclei and FSHR expression. Representative images are shown (*n* = 3, Scale bar = 400 μm).

Follicular fluid was collected from follicles smaller than 3 mm and 3–8 mm in diameter. High-performance liquid chromatography was used to detect arginine levels in the follicular fluid. Table 2 shows detectable levels of arginine in both types of follicular fluid. The arginine concentration in fluid from follicles <3 mm (3.5499 ± 0.0955) was significantly higher than that in fluid from follicles 3–8 mm (2.7016 ± 0.6555). Granulosa cells were collected from ovarian follicles 3–8 mm in diameter for culture. FSHR was specifically expressed in granulosa cells. As shown in Figure 1, DAPI staining resulted in blue fluorescence in cell nuclei, while FSHR expression in the cytoplasm exhibited red fluorescence. This confirmed that the isolated and cultured cells were porcine ovarian granulosa cells suitable for subsequent experiments.

**Table 2:** Determination of arginine content (mM) in follicular fluid of follicles smaller than 3 mm in diameter by liquid chromatography.

Amino acid	Follicle diameter	Number of samples	Mean ± S. E. M
Arginine (mM)	Smaller than 3 mm	45^i^	3.5499 ± 0.09554
Arginine (mM)	3-8 mm	45	2.7016 ± 0.6555^**^

** was significantly different (*p* < 0.01).

### 3.2 The effect of arginine on the proliferation of porcine ovarian granulosa cells

**Figure 2:**
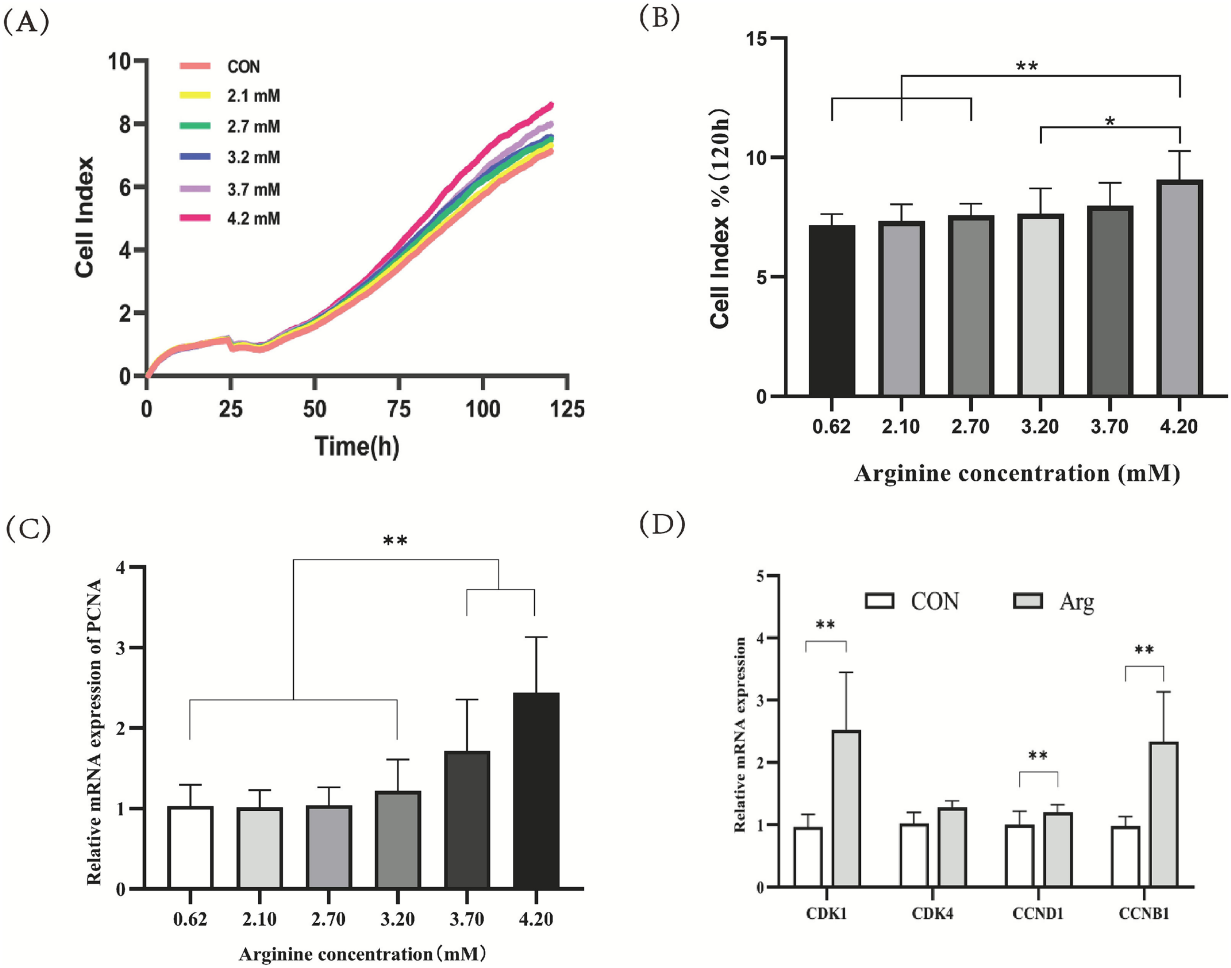
Effects of arginine on proliferation of porcine ovarian granulosa cells. **(A)** RTCA proliferation curve (*n* = 4); **(B)** RTCA proliferation rate (*n* = 4); **(C)** Gene expression of porcine ovarian granulosa cell proliferation gene (*n* = 9); **(D)** The levels of mRNA expression for *CDK1* and *CCNB1* are presented here (*n* = 9). *The difference was significant (*p* < 0.05); ** was significantly different (*p* < 0.01) “CON” was “CONTROL group,” “Arg” was “Arginine group”.

Based on the arginine concentration results measured in Section 3.1 (arginine concentrations in 3–8 mm follicles), primary porcine ovarian granulosa cells were cultured in DMEM/F12 basal complete medium containing 0.62 mM arginine as the control group. Simultaneously, five treatment groups with different arginine concentrations (2.1, 2.7, 3.2, 3.7, and 4.2 mM) were established for culture. Results (Figures 2A,B) showed that the 120-h cell proliferation rate in the 4.2 mM arginine group was significantly higher than that in the control group, the 2.1 mM arginine group, and the 2.7 mM arginine group (*p* < 0.01); while the 4.2 mM group showed significantly higher proliferation than the 3.2 mM group (*p* < 0.05). The detection of mRNA expression levels for genes associated with ovarian granulosa cell proliferation was also performed. RT-qPCR results (Figure 2C) showed that, compared to the control group, mRNA expression levels of the *PCNA* gene, which is related to cell proliferation, were significantly higher in both the 3.7 mM and 4.2 mM Arg groups (*p* < 0.01). *PCNA* gene mRNA expression levels showed a dose-dependent increase with rising arginine concentrations. As shown in Figure 2D, the results indicate that the expression levels of mRNA for *CDK1* and *CCNB1* were significantly higher in the arginine-treated group than in the control group (*p* < 0.01). However, no significant differences were observed in the expression of *CDK4* and *CCND1*.

### 3.3 Effects of arginine on apoptosis in porcine ovarian granulosa cells

**Figure 3:**
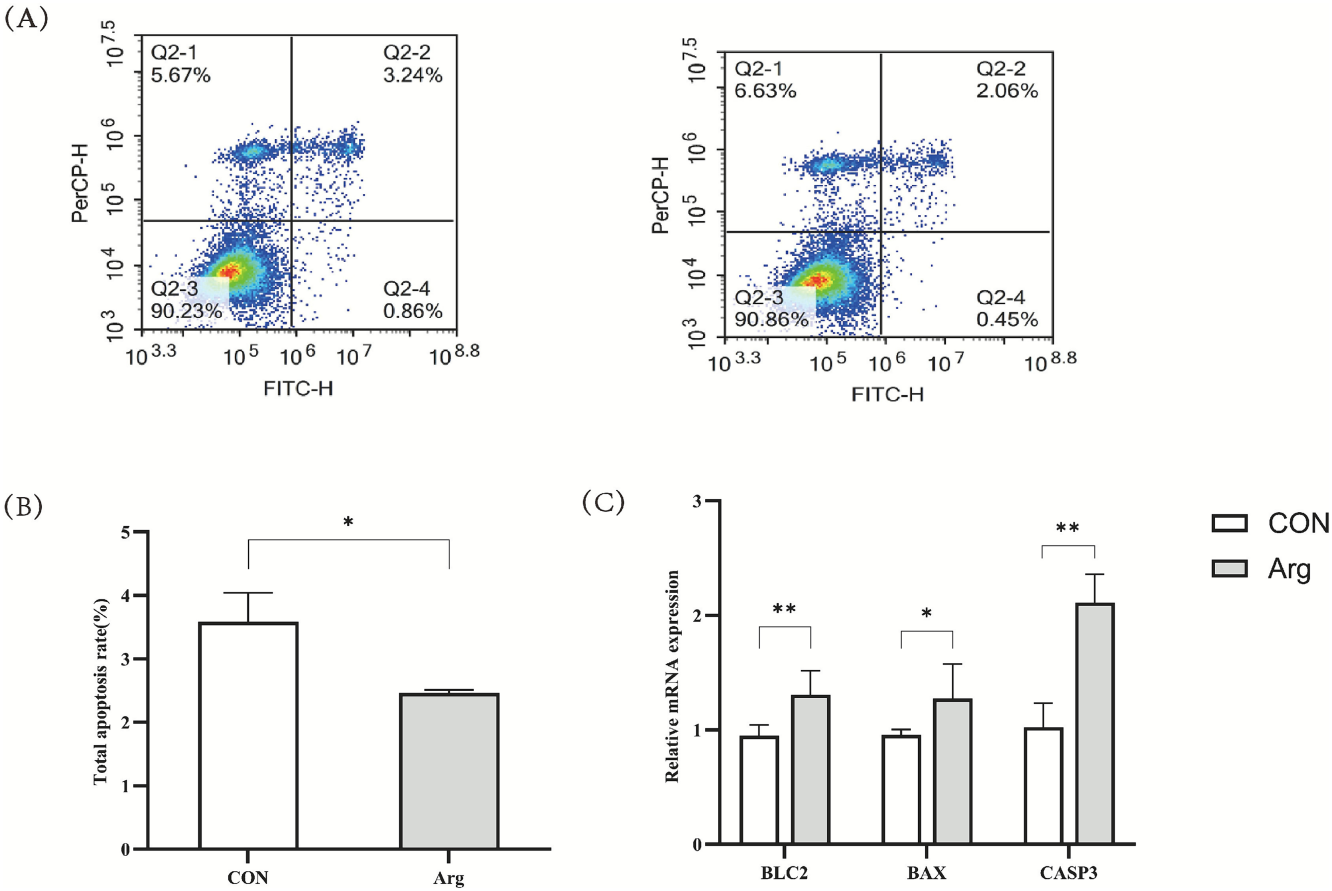
Effects of arginine on apoptosis of porcine ovarian granulosa cells. **(A)** Flow cytometric plots for detecting apoptosis by Annexin V and PI staining; **(B)** Apoptosis rate of porcine granulosa cells by flow cytometry (*n* = 4); **(C)** Gene expression of genes related to porcine granulosa cell apoptosis (*n* = 9). * significantly different (*p* < 0.05) and ** was significantly different (*p* < 0.01). “CON” was “CONTROL group,” “Arg” was “Arginine group”.

The results of the flow cytometry analysis (Figures 3A–C) indicate that the late apoptosis rate in the 4.2 mM Arg group was extremely significantly lower than in the control group (*p* < 0.01), while the early and total apoptosis rates were significantly lower than in the control group (*p* < 0.05). RT-qPCR results showed that mRNA expression levels of the *BCL2* and *CASP3* genes were significantly higher in the 4.2 mM Arg group than in the control group (*p* < 0.01), and that the *BAX* gene expression level was significantly higher than in the control group (*p* < 0.05).

### 3.4 The effect of arginine on steroid hormone synthesis

**Figure 4:**
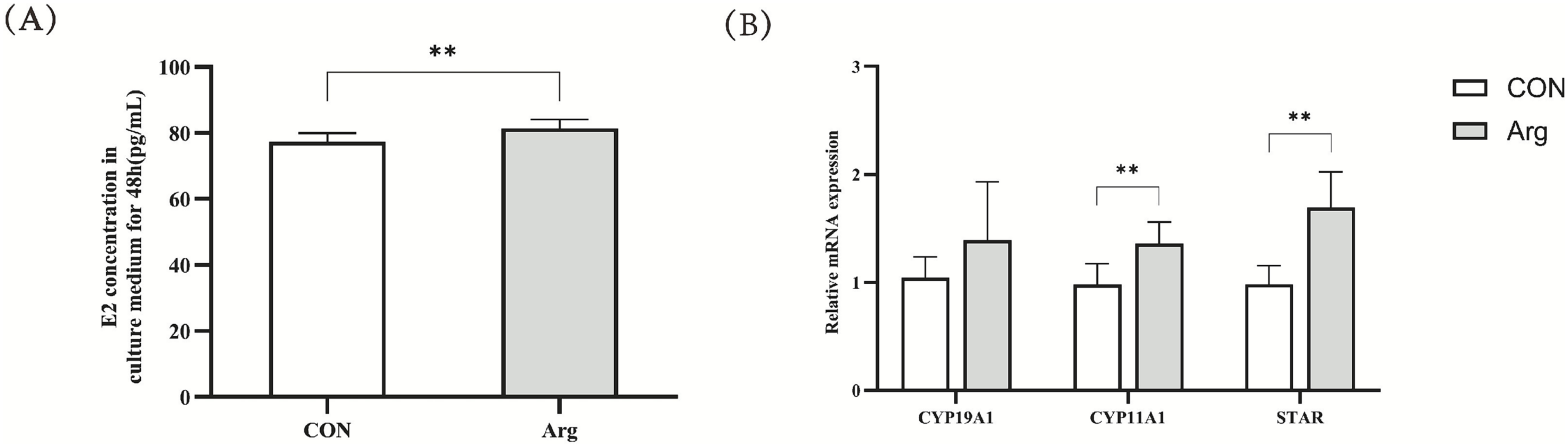
Effect of arginine on steroid hormone synthesis in granulosa cells **(A)**: The level of estradiol in the culture medium from 48 h (*n* = 10); **(B)**: the relative expression of mRNA of granulosa cell sterol hormone synthesis related genes. Data are presented as mean ± SEM from three independent biological replicates (*n* = 9). ** was significantly different (*p* < 0.01). “CON” was “CONTROL group,” “Arg” was “Arginine group”.

The results shown in Figure 4 indicate that, after 72 h of *in vitro* culture, the primary porcine ovarian granulosa cells were placed in either the arginine-treated group (4.20 mM Arg) or the control group (0.62 mM CON), both using a DMEM/F12-based complete medium. The results show that arginine significantly promotes E2 secretion (*p* < 0.01). Fluorescent quantitative PCR results show that mRNA expression levels of *CYP11A1* and *STAR* were significantly higher in the Arg group than in the CON group (*p* < 0.01). However, the difference in the mRNA expression level of *CYP19A1* between the two groups was not significant.

### 3.5 Arginine-mediated STARD3 and STARD6 promote steroid hormone synthesis in porcine ovarian cells

**Figure 5:**
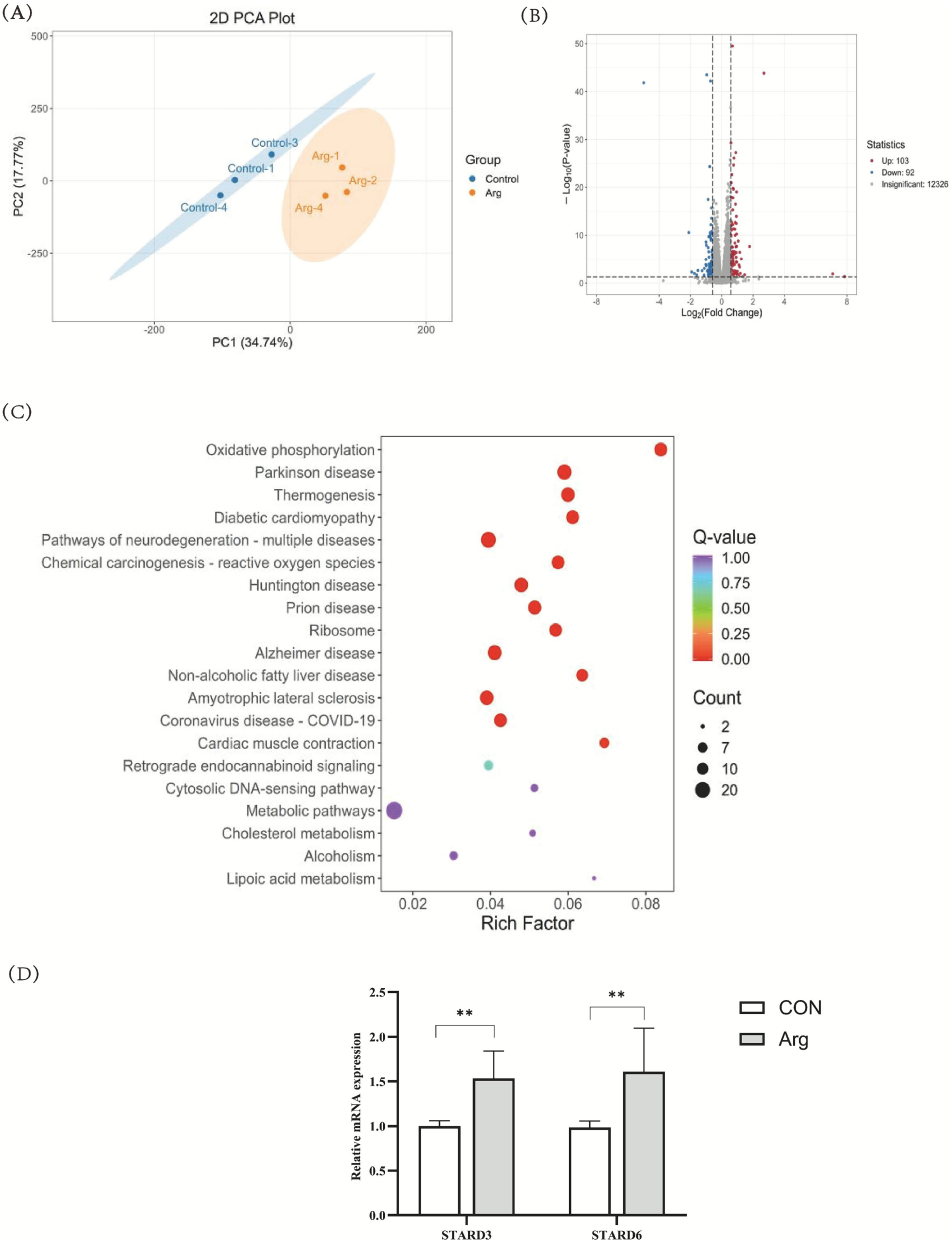
Effect of arginine on the transcriptomic profile of porcine ovarian granulosa cells analyzed by RNA-seq (*n* = 3). **(A)** Principal component analysis (PCA) 2D diagram of porcine ovarian granulosa cells. **(B)** Volcano map. **(C)** Scatter plot of KEGG enrichment results. **(D)** Validation of mRNA levels of significantly differentially expressed genes (DEGs) by quantitative real-time PCR. ** was significantly different (*p* < 0.01). “CON” was “CONTROL group,” “Arg” was “Arginine group”.

To further investigate the molecular mechanisms underlying arginine’s effects on ovarian granulosa cells, we performed transcriptome sequencing on porcine ovarian granulosa cells from the control group (0.62 mM Arg) and the arginine group (4.2 mM Arg). Principal component analysis (PCA) revealed a significant separation trend between control and arginine group samples (Figure 5A). Differential expression analysis revealed that, compared to the control group, 195 genes exhibited significant differential expression (|log₂FoldChange| > 1, *p* < 0.05) in the arginine-treated group, comprising 103 upregulated genes and 92 downregulated genes (Figure 5B). These differentially expressed genes demonstrated pronounced expression changes between the two groups. To further analyse the functional distribution of differentially expressed genes, KEGG pathway enrichment analysis was performed on these genes. Enrichment results indicated that differentially expressed genes were predominantly distributed across multiple metabolism-related pathways, with the Cholesterol metabolism pathway being one of the significantly enriched pathways. Within cholesterol metabolism pathways, multiple differentially expressed genes were detected (Figure 5C). Among these, *STARD3* and *STARD6* were identified as significantly differentially expressed genes, exhibiting an upregulation trend in the arginine-treated group relative to the control group. qRT-PCR was used to validate the mRNA levels of two significantly differentially expressed genes involved in cholesterol transport in porcine ovarian granules. In comparison with the CON group, Arg treatment resulted in highly significant upregulation of *STARD3* and *STARD6* (Figure 5D) (*p* < 0.01), consistent with the RNA-seq analysis results. This validation serves to demonstrate the high reliability of the RNA-seq analysis. Transcriptome sequencing results indicate that arginine may regulate cholesterol metabolism and transport through the expression of *STARD3* and *STARD6* genes, thereby influencing the function of follicular granulosa cells.

## 4 Discussion

In the contemporary realm of swine production, the quest for enhanced reproductive performance occupies a pivotal position in the pursuit of optimized economic efficiency. The reproductive outcomes of sows, encompassing both quantity and quality of piglets, exert a direct and substantial influence on the profitability of the industry as a whole. The ovaries, as the fundamental component of the sow’s reproductive system, play a pivotal role in successful breeding. Their proper functioning is imperative for optimal reproductive outcomes. Granulosa cells are pivotal to several crucial processes within the ovary, including follicular development, hormone secretion, and oocyte maturation. These cells engage in dynamic signaling and material exchange with oocytes, thereby exerting a profound influence on key reproductive parameters such as ovulation rates, fertilization rates, and the quality of early embryonic development. Consequently, granulosa cells are instrumental in determining PSY (pig performance index), a pivotal metric that serves as a benchmark for evaluating the efficiency of swine production systems (2).

As a fundamental element of the microenvironment conducive to oocyte development, alterations in the amino acid composition and content of porcine follicular fluid exert a direct influence on follicular development, oocyte maturation, and the viability of early embryos (26). The results of the present study have determined the physiological concentration of arginine in follicular fluid from follicles measuring 3–8 mm in diameter. This determination was made by analysing the amino acid content of follicular fluid in pigs. The study established that the arginine content in follicular fluid demonstrates a certain range of variation among different individuals. Furthermore, it was determined that the amino acid concentration in follicular fluid changes with variations in nutritional levels (11). The experiment established five distinct concentrations of arginine supplementation, three of which exceeded and two of which fell below physiological levels, with the objective of investigating the impact of varying concentrations of arginine on the function of porcine ovarian granulosa cells. A study was conducted to determine the effect of arginine on the proliferation of porcine ovarian granulosa cells. The results of the experiment indicated that the cell proliferation rate in the group with a concentration higher than physiological levels (4.20 mM Arg group) was significantly greater than that in the 0.62 mM CON group. Furthermore, the mRNA expression level of PCNA in the 4.20 mM Arg group was significantly higher than that in the 0.62 mM CON group. These findings suggest that arginine can promote the proliferation of porcine ovarian granulosa cells. This finding is consistent with previous research on the effects of arginine on ovarian granulosa cells in pigs and cattle (10, 21). However, the findings of our investigation indicate that the proliferation efficiency attained its zenith in the 4.20 mM Arg group, a concentration that surpasses physiological concentrations. Moreover, the validity of this promotional effect has been substantiated at the gene expression level. PCNA (proliferating cell nuclear antigen), a key marker of cell proliferation (27), showed increased mRNA expression. The results of this study indicate that arginine facilitates the orderly progression of the cell cycle by regulating key molecules associated with proliferation, such as *PCNA* (proliferating cell nuclear antigen) and *CDC42* (cell division cyclin). The upregulation of these genes may facilitate the smooth transition of granulosa cells from the G1 phase to the S phase for DNA replication, thereby accelerating the cell division and proliferation process (15, 28). The primary findings of this study further confirm the role of arginine in promoting the proliferation of porcine ovarian granulosa cells, providing a more robust theoretical foundation for the application of arginine in the field of swine reproduction.

To the best of our knowledge, no studies have previously reported on the effects of arginine on apoptosis in porcine ovarian granulosa cells. The present experiment employed the technique of flow cytometry analysis and found that the total apoptosis rate in the Arg group was significantly lower than that in the CON group. The results of the fluorescent quantitative PCR analysis revealed that the mRNA expression levels of the BCL2 and CASP3 genes were significantly higher in the Arg group than in the CON group. Meanwhile, the mRNA expression of the BAX gene was markedly elevated. BCL2 is an anti-apoptotic protein, and BAX is a pro-apoptotic protein. The ratio of these two proteins largely determines a cell’s sensitivity to apoptotic stimuli (29). Under normal circumstances, increased expression of BCL2 has been shown to inhibit apoptosis, while high expression of BAX has been demonstrated to promote it. However, the results of this study indicate that both of these factors were elevated simultaneously, suggesting that the regulatory mechanism of apoptosis by arginine is relatively complex. In summary, there is evidence to suggest that arginine may inhibit apoptosis to a certain degree by modulating intracellular apoptotic signaling pathways. CASP3 is a pivotal executor protein in the process of apoptosis. However, research has shown that elevated levels of CASP3 expression are counterintuitively associated with decreased rates of apoptosis. This phenomenon may be due to the influence of arginine on CASP3 activation or the presence of compensatory mechanisms that counteract its pro-apoptotic effects (20). An exhaustive inquiry into the regulatory mechanisms by which arginine modulates the expression of these apoptosis-related genes, as well as their intracellular interactions, is imperative for elucidating arginine’s role in ensuring the survival and function of porcine ovarian granulosa cells.

In the regulatory processes governing female mammalian reproduction, ovarian granulosa cell proliferation and estrogen synthesis play a central role in follicular development. Estradiol has been shown to maintain granulosa cell function by activating membrane and nuclear receptor pathways in synergy with follicle-stimulating hormone (5). Recent research has demonstrated that the amino acid arginine can upregulate the expression of *STAR* and *CYP19A1* mRNA. *STAR* functions as the rate-limiting factor for cholesterol transport across the mitochondrial membrane. Increased STAR expression directly promotes pregnenolone production, thereby providing precursor substances for estrogen synthesis (2, 22). CYP19A1 catalyzes the conversion of androgens to estrogens (30), establishing a core pathway in which arginine promotes estrogen secretion. This indicates that arginine enhances hormone secretion in porcine ovarian granulosa cells, consistent with the findings of previous studies (21). Disturbances in serum cholesterol levels can affect steroidogenesis and reproductive functions (31). The present study provides evidence that the amino acid arginine may facilitate the conversion of cholesterol to estrogens by transcriptionally activating the key enzymes STAR and CYP19A1. Arginine activates critical molecules at pivotal nodes of the steroid synthesis pathway, thereby significantly enhancing the efficiency of estrogen biosynthesis. Consequently, it promotes granulosa cell proliferation through autocrine and paracrine mechanisms. However, the precise molecular mechanisms require further validation.

The present study employed transcriptome sequencing to procure high-quality data. Each sample yielded 8 Gb of clean data. The percentage of Q30 bases in valid reads attained 94% or higher, and the proportion of sequences mapped to the genome surpassed 96%. This indicates that the sequencing depth and quality have met the experimental requirements, thereby providing a robust foundation for subsequent accurate analysis of gene expression changes. Gene expression level analysis primarily employed principal component analysis using FPKM values. The gene expression levels between replicates within each sample of the control and treatment groups exhibited high correlation coefficients, thus demonstrating excellent reproducibility between replicates. A differential gene expression analysis was conducted, revealing 195 differentially expressed genes between the arginine-treated and control groups. Of these, 103 were up-regulated and 92 were down-regulated. These differentially expressed genes may participate in a variety of biological processes, including cell proliferation, metabolism, and signal transduction. Subsequent studies will entail functional annotation and classification of these differentially expressed genes, thereby facilitating a more profound understanding of the specific molecular mechanisms through which arginine regulates cellular functions. The present study found that the cholesterol metabolism pathway was significantly enriched. Furthermore, two key genes, *STARD3* and *STARD6*, were identified as being involved in the process. Cholesterol plays a pivotal role in follicular development. As a precursor for steroid hormone synthesis, it is essential for maintaining the normal function and hormone secretion of granulosa cells within the follicle (32). Concurrently, the mRNA levels of two genes with significantly differential expression (*STARD3* and *STARD6*) were validated by real-time quantitative PCR. The results of this analysis were consistent with the transcriptome sequencing analysis. Furthermore, arginine (Arg), a functional amino acid, plays a role in protein synthesis and influences cellular metabolism and the supply of hormone synthesis precursors by regulating the nitric oxide pathway, cholesterol transport, and mitochondrial function (33). Therefore, the findings of this study suggest that arginine can regulate cholesterol metabolism and transport by influencing the expression of *STARD3* and *STARD6*. This affects the function of follicular granulosa cells and reproductive performance in pigs.

Despite the valuable findings yielded by this study, certain limitations remain. Firstly, it is crucial to note that the present study was conducted under *in vitro* cell culture conditions, which differ from the complex physiological environment *in vivo*. Ovarian tissue within the body is subject to meticulous regulation by an array of hormones, cytokines, and intercellular interactions (34). The role of arginine in the body may be more complex. Our findings lay the foundation for future research in this field. We have discovered that the supplementation of arginine, at concentrations surpassing physiological levels, promotes the proliferation of porcine ovarian granulosa cells and stimulates steroid hormone secretion. This approach, when combined with in vivo experiments, such as the incorporation of arginine into the diets of gilts, exerts a profound influence on granulosa cell function by augmenting arginine concentrations within follicular fluid. This, in turn, fosters oocyte development and further enhances sow reproductive performance. This study has identified an association between the cholesterol metabolism pathway and arginine treatment. However, the initiation and maintenance of reproductive functions normally depend upon gonadotropins that in turn cause the production of steroids, further investigation is required to elucidate the manner in which alterations in cholesterol metabolism specifically influence physiological processes such as granulosa cell proliferation and hormone synthesis in follicles. Future research endeavors may endeavor to integrate multi-omics technologies, including proteomics and metabolomics, to undertake a comprehensive investigation into the regulatory mechanisms of arginine on porcine follicular granulosa cells. Concurrently, techniques such as gene knockout and overexpression can serve to validate the roles of key genes (*STARD3* and *STARD6*) in arginine-mediated cholesterol metabolism and granulosa cell function. This, in turn, will provide stronger theoretical support for the precise application of arginine to enhance sow reproductive performance.

## 5 Conclusion

In summary, this study demonstrates that arginine supplementation at a concentration of 4.2 mM significantly promotes proliferation and inhibits apoptosis in porcine ovarian granulosa cells, while enhancing their *in vitro* oestradiol secretion capacity. Transcriptomic analysis further indicates that arginine regulates steroid hormone synthesis by modulating cholesterol metabolism, with *STARD3* and *STARD6* identified as key regulatory targets. These findings underscore the role of arginine as an important nutritional regulator of theca cell function and elucidate its mechanism in supporting ovarian steroidogenesis. From a practical perspective, these results provide a theoretical basis for the rational application of arginine to improve swine reproductive performance.

## Data Availability

The original contributions presented in the study are publicly available. This data can be found: the data has been uploaded to the SRA of NCBI link: https://www.ncbi.nlm.nih.gov/bioproject/PRJNA1379295.
